# Abnormal right atrial performance in surgically repaired Tetralogy of Fallot: the German Competence Network for Congenital Heart Defects investigators

**DOI:** 10.1186/1532-429X-17-S1-Q84

**Published:** 2015-02-03

**Authors:** Shelby Kutty, Quanliang Shang, David Danford, Michael Steinmetz, Andreas Schuster, Titus Kuehne, Philipp B Beerbaum, Samir Sarikouch

**Affiliations:** 1 University of Nebraska Medical Center, Chiildren's Hospital and Medical Center, Omaha, NE USA; 2 University Medical Center, Göttingen, Germany; 3 German Heart Institute, Berlin, Berlin, Germany; 4 Hanover Medical University, Hannover, Hannover, Germany

## Background

The purpose of the study was to test the hypothesis that right atrial (RA) performance is abnormal in surgically repaired tetralogy of Fallot (TOF).

## Methods

TOF patients were prospectively enrolled for same day cardiovascular magnetic resonance (CMR), echocardiography and exercise stress test following a standardized protocol in 14 participating centers of the German Competence Network for Congenital Heart Defects. Peak longitudinal RA strain (RAS), peak longitudinal right ventricular strain (RVS), RA and RV enddiastolic volumes (EDV, indexed to body surface area) and ejection fraction (EF) were measured. RAS and RVS were obtained from fourchamber cine (6segment model) using CMRfeature tracking (FT, CPAMR, Tomtec).

## Results

The cohort consisted of 196 subjects: 171 patients with repaired TOF (94 male, mean age 18±8 yrs) and 25 normal controls (13 male, 35±14years). Fiftyfour TOF patients had follow up studies at 1 year, yielding 225 TOF examinations. All subjects were in sinus rhythm. RA EDV, RAS, RVS, RA EF, and RV EF in TOF were 60 ± 19 ml/m2, 14±6%, 13±4%, 33±10% and 51±8%; and differed significantly from the respective indices in controls: 34±9 ml/m2, 30±10%, 21±4%, 51±9% and 64±9% (all p<0.001). RA EDV and RAS correlated directly with RVS (both p<0.001). RA EDV was higher in older TOF patients, while RAS did not increase with age. RA EDV, but not RAS, correlated positively with echoderived RV systolic pressure. Neither RA EDV nor RAS was associated with tricuspid regurgitation grade or VO2 max. Positive correlation was observed for RV EDV with RA EDV (p=0.035), and a trend toward negative correlation with RAS (p=0.09).

## Conclusions

RAS, RA EDV and RA EF are all generally abnormal in TOF. Because they correlate with other functional RV indices, these abnormalities may represent RA diastolic burden from chronic RV dysfunction in TOF. The young age of the study cohort might explain the absence of RAS correlation to tricuspid regurgitation and VO_2_ max.

## Funding

German Competence Network for Congenital Heart Defects.

